# Playing the political game: the coevolution of institutions with group size and political inequality

**DOI:** 10.1098/rstb.2022.0303

**Published:** 2023-08-14

**Authors:** Simon T. Powers, Cedric Perret, Thomas E. Currie

**Affiliations:** ^1^ Edinburgh Napier University, Edinburgh EH10 5DT, UK; ^2^ University of Exeter, Penryn TR10 9FE, UK

**Keywords:** institutions, cooperation, hierarchy, punishment, political inequality

## Abstract

All societies need to form institutional rules to regulate their social interactions. These specify what actions individuals should take in particular situations, and what sanctions will apply if individuals violate these rules. However, forming these institutional rules involves playing a political game—a process of negotiation between individuals that is costly and time-consuming. Intuitively, this cost should be expected to increase as a group becomes larger, which could then select for a transition to hierarchy to keep the cost of playing the political game down as group size increases. However, previous work has lacked a mechanistic yet general model of political games that could formalize this argument and test the conditions under which it holds. We address this by formalizing the political game using a standard consensus formation model. We show that the increasing cost of forming a consensus over institutional rules selects for a transition from egalitarian to hierarchical organization over a wide range of conditions. Playing a political game to form institutional rules in this way captures and unites a previously disparate set of voluntary theories for hierarchy formation, and can explain why the increasing group size in the Neolithic would lead to strong political inequality.

This article is part of the theme issue ‘Evolutionary ecology of inequality’.

## 1. Introduction

All societies, from hunter–gatherers to modern liberal democracies, are governed by institutional rules [1,2] that help to coordinate individuals, promote cooperative behaviour, and resolve social dilemmas [3–5]. Many hunter–gatherer societies have rules that regulate the conditions under which individuals should give food to others [6]. For example, the !Kung Bushmen have a rule that says that the owner of the first arrow that penetrates the animal controls distribution after a cooperative hunt [7]. Likewise, societies that have developed irrigation systems have rules that specify how much water an individual farmer may take and when, and how much they should contribute to maintaining the system [2,8]. These rules are often enforced by systems of monitoring and sanctioning [5]. These range from gossip, ridicule and ostracism in hunter–gatherers [9], through to third-party punishment by the state in liberal democracies. Economics has long studied the effects that institutional rules have on incentivising cooperative social behaviour among self-interested agents (e.g. [1,3,10]). Indeed, it is even argued that the main determinant of whether a nation is poor or prosperous is the type of institutional rules governing the society, and whether these successfully incentivise cooperative division of labour and trade [11].

Perhaps the most prominent pattern in human history has involved changes in the institutional arrangements that govern human socio-political organization. As societies increased in size following the origin of agriculture and the Neolithic Demographic Transition [12], they switched from egalitarian to hierarchical organization [9,13,14]. Here, we consider hierarchy as a skewed influence in decision-making, such that a minority of individuals—leaders—have a disproportionate influence in the decision a group takes. Analyses of a global, long-term historical and archaeological database show that the number of hierarchical levels of organization, and the complexity, of other governance institutions increased as the population size of polities also increased [15]. One potential explanation for this relationship between hierarchical organization and the size of groups relates to the fact that as group size increases, coordination problems such as deciding on institutional rules become more difficult to solve—a general phenomenon known as scalar stress [16]. Hierarchy is potentially a means by which groups can overcome these problems and be able to make and enforce decisions more efficiently. For example, in the Enga of Papua New Guinea, clans, which generally incorporate a few hundred individuals, make decisions about how to act in relationships with other groups through whole clan meetings. These meetings require consensus to be reached among all male members of the clan [17]. In modern countries, with populations in the millions, such consensus would be almost impossible, and only a small subset of individuals act as political leaders, who formally spend their time negotiating over and deciding institutional rules.

In this paper, we examine the hypothesis that as group size increased during the Neolithic, the process of discussing, negotiating, and deciding on institutional rules became more and more costly for egalitarian groups. This could then select for a transition to hierarchical organization to allow groups to continue to grow in size while reducing the cost of rule formation [1,18]. We use ‘group size’ here to mean the size of the unit engaged in collective action. While the empirical relationships between hierarchical organization and group size are well established, a key research challenge in fields such as anthropology, archaeology, political science, and economic history, is understanding how institutional rules are formed and evolve. Yet despite the importance of these issues, relatively little work has been done in these disciplines in formally modelling the processes of institutional rule formation. For example, cultural group selection models have considered how certain institutional rules might give one group an advantage over another [19], but have typically not considered how institutional rules are formed within a single group.

Recently, some researchers have taken a game theoretic perspective to modelling the evolution of institutional rules [20–22]. This has drawn on work from economics that proposes a meta-model of institutional rule formation [10,23]. This meta-model separates social interactions within a group into an economic game and a political game [24]. In the economic game, individuals have social interactions that directly affect their material pay-off (calorie intake, size of shelter etc.). Examples include choosing whether to join a cooperative hunting party, how much food to share with other group members, how much water to take from an irrigation system, and whether or not to pay taxes. This is the standard type of game studied in evolutionary anthropology, archaeology, and biology, and might be modelled as a public goods or Prisoner’s Dilemma game, for example. However, in addition to this, group members also engage in a political game that sets the rules of this economic game. For example, how much water should each group member take from an irrigation system, how will this be monitored, and what will the sanctions be for taking more than the rules permit? The rules that result from the political game are known as the institutional rules, and determine the effective pay-off matrix (or more formally, the game form, [23]) of the economic game. Consequently, they determine the extent to which self-interested individuals who are trying to maximize their material pay-off will cooperate [3,5].

Political games essentially involve individuals discussing, negotiating over, and deciding about how people should behave in their economic interactions [24]. This most obviously occurs in liberal democracies and states such as ancient Athens [25], where formal assemblies of representatives of the rest of the population debate over codified laws that govern economic interactions [10]. However, at the other end of the scale, hunter–gatherer societies spend a vast amount of time discussing the rules of their society and whether individuals are complying with them [9]. For example, in the Mae Enga, clan meetings are used to reach consensus about relationships with other groups (warfare or trade), while other activities and situations are discussed within the lower level of sub-clans. In Lamalera, Indonesia, an annual ceremony (Tobo Namã Fata) precedes the whaling season [26]. The ceremony involves the boat owners, harpooners, and master carpenters who have to coordinate their activities and receive shares of a successful hunt. They are joined at the ceremony by clan leaders, and the attendants discuss any problems or conflicts that occurred in the previous whaling season, and potentially establish new norms to clarify situations in the future. The political game can thus be more or less formal. Crucially, it may involve all or only a subset of group members. In egalitarian societies, most individuals might take part in negotiating over rules, while in hierarchical societies only elites (e.g. the leader and their kin) might take part. In either case, we would expect individuals to try and influence the political game to produce rules that will benefit themselves. In other words, individuals will have a strategy in the political game, i.e. a preference for the rules that they would like to see be implemented. Note that we do not need to assume that individuals are fully rational and completely aware of the consequences of their decisions to be able to play a political game in this way. Trial-and-error, or cultural evolution through pay-off-biased social learning [27], will over time lead individuals to adopt strategies in the political game that produce rules which increase their material pay-off in the economic game.

However, previous work has largely ignored the cost to individuals of playing the political game. Playing a political game—bartering and negotiating over rules—clearly carries an opportunity cost for the individuals involved. Simply put, time spent negotiating and trying to convince others is time that could otherwise be spent on productive economic activities such as hunting, fishing or farming (i.e. playing the economic game for material pay-off). To be able to address this, we need a model of the political game that is general enough to capture many ways in which rules are created, debated, and modified across different societies and contexts, while being mechanistic enough to demonstrate how the cost of rule formation increases with group size. In previous work, political games have generally been modelled very abstractly in a way that does not allow the relationship between their cost and group size to be analysed. Some models have only considered institutional rules as group-level traits that change by unspecified (effectively random) processes within groups followed by group competition [19,28]. Other models have captured the political game within a group as simply taking the mean preference of the group members [20,29], or taking a majority vote [21]. None of these are detailed enough to show how the cost of rule formation scales with group size.

Here, we overcome this limitation by using a standard consensus formation model [30,31] to capture the political game. In a consensus formation model, individuals start with an initial opinion, and then modify this based on interactions with other individuals until a consensus is reached where the variance of their opinions is less than some threshold. How much individuals sway the consensus towards their own opinion depends on their *influence* relative to the influence of other individuals—the consensus is closer to the opinion of individuals with higher influence. Variation in influence can be owing to endogeneous differences in personality traits such as persuasiveness, stubbornness and talkativeness [30–32], or exogeneous differences in wealth or social network size [33]. In an egalitarian group, each individual would have roughly the same influence, while in a hierarchical group leaders would have a higher influence than followers. Crucially, the time it takes to reach consensus depends on the distribution of influence—if all individuals have similar influence, i.e. the group is egalitarian, then it will take longer to reach consensus than if a few individuals have much higher influence, i.e. the group is hierarchical [30,34]. Moreover, previous work has shown that consensus time increases with group size, and at a faster rate in egalitarian compared to hierarchical groups [34].

By considering the influence of an individual as an evolvable trait, and considering the time to reach consensus as an opportunity cost of playing the political game that is subtracted from individual fitness, we can demonstrate conditions under which evolution will lead to egalitarian or hierarchical organization in response to the demands of rule formation. To do this, we build on an existing model of the coevolution of institutions, demography and cooperation during the Neolithic [20]. This demonstrated conditions under which institutional rules for punishment coevolved with group size to support the transition from small-scale to large-scale cooperative groups. However, it considered a fixed cost of playing the political game, which was an exogeneous parameter. Here, we replace that abstract model of the political game with a consensus formation model, and also allow individuals to evolve their influence.

## 2. Model description

Our model builds upon the model of the coevolution of institutions, demography and large-scale cooperation presented in [20]. In that model, individuals live on a number *N*_p_, of resource patches connected by migration, and can be either social or asocial. Social individuals agree to form an institution on their patch, and then play a public goods game regulated by the institutional rules. Asocial individuals, on the other hand, do not take part in institution formation or the public goods game. The inclusion of asocial individuals allows the model to demonstrate the origin of an institution, by showing conditions under which self-interested individuals would choose to form an institution.

The institutional rules, *p*_*j*_, specify how much of the public good generated by social individuals on their patch *j* is invested into monitoring and punishment of defectors—individuals who do not cooperate in the public goods game. This share of the public good is used to pay for monitoring and sanctioning. This corresponds to situations where groups can create institutional rules that incentivise individuals to monitor and sanction others [35], rather than monitoring and sanctioning being altruistic acts (i.e. sanctioning is not altruistic as in [36], but provides direct benefits to those doing it [5,24]). For example, Ostrom describes how in the Spanish *huerta* irrigation systems, some group members are nominated to act as monitors by their peers, and are further incentivised to catch defectors by being able to keep a third of the fine levied on any defector they catch [2]. So rather than being individually costly, as in classic models of peer-punishment, these institutional rules turn sanctioning into a profitable activity for some individuals. This issue is discussed in more detail in the presentation of the original model [20]. Further empirical examples of institutional rules covering the costs of monitoring and sanctioning are discussed in [8]. While we do not explicitly model the individual monitoring and sanctioning role here (but see [37] for such a model), our model captures this role of institutional rules in a high-level manner, allowing us to focus our analysis on the political game.

The remaining share of social individuals’ public good, 1 − *p*_*j*_, is invested into producing technology that increases the carrying capacity of all social individuals on the patch, e.g. an irrigation system. In [20], the institutional rules *p*_*j*_ were formed by taking the mean preference of all social individuals on the patch, and all social individuals payed a fixed opportunity cost *I* for doing this compared to asocials. This represented the political game.

The results were that if patches started off at a small size (around 20 individuals) then social individuals could invade a population of asocials, and evolved to create institutional rules that selected for high levels of cooperation. Specifically, individuals evolved their own preferences for the rules to invest most of their public good into technology that raised their carrying capacity, while investing just enough (around 15–20 per cent) into monitoring and punishment to prevent defection in the public goods game from being individually advantageous. This occurred because individuals in patches with these kinds of rules had more offspring compared to asocials, and compared to individuals on patches with other institutional rules that invested either so much into monitoring and punishment that they lost the benefits of producing technology, or so little that social defectors outcompeted cooperators. Groups with these optimal institutional rules then grew in size (up to hundreds of individuals) owing to technology increasing their carrying capacity, capturing the demographic transition from small to large groups that occurred during the Neolithic.

We now go on to provide a complete specification of the model, focusing on how we reimplement the political game with a consensus formation model, and on how we now allow individuals to evolve their influence, and hence the level of hierarchy on their patch.

### (a) Life cycle and population structure

The life cycle and population structure follows that in [20]. The population is subdivided into a finite number, *N*_p_, of resource patches, that are connected by migration following Wright’s finite island model of dispersal [38]. The life cycle of individuals consists of discrete and non-overlapping generations, as follows: (i) local interactions occur on each patch, with social individuals forming institutional rules and playing a public goods game in which they can either cooperate or defect; (ii) each individual *i* on patch *j* has a Poisson distributed number of offspring that survive to adulthood, with the mean of the distribution being determined by the local social interactions and resource abundance (defined explicitly below); (iii) adults of the previous generation perish; and (iv) each individual of the descendant generation migrants to a randomly chosen patch with probability *m*, or otherwise remains on its natal patch.

### (b) Evolving traits

Individuals carry three cultural traits that are transmitted vertically from parent to offspring. This kind of transmission of cultural traits such as preferences and values from parents to offspring is common in both hunter–gatherer groups [39] and modern societies [40]. The first trait, *τ*_*ij*_, is discrete and determines the social type of the individual. That is, whether the individual is *asocial*, *τ*_*ij*_ = a (does not take part in institution formation, receives no public good, and are not sanctioned), a social *cooperator*, *τ*_*ij*_ = c (takes part in institution formation and cooperates in the public goods game), or a social *defector*, *τ*_*ij*_ = d (takes part in institution formation but defects in the public goods game by not contributing). Mutation occurs on this trait with probability $\mu_{\tau }$ by switching to one of the other two values chosen at random.

The second trait, *p*_*ij*_, determines the intrinsic preference that the individual has for the institutional rules, specifically, the proportion of the public good on its patch that it thinks should be used to pay for the monitoring and punishment of defectors, as opposed to invested in technology. This trait is continuous in the range [0, 1].

In this paper, we add a third trait, *α*_*ij*_, which determines the influence of the individual in the consensus formation process (political game). This is also continuous in the range [0, 1]. A larger value of *α*_*ij*_ means that the individual is more stubborn and persuasive when discussing with other individuals about what the institutional rules should be [30,31,34]. For both *p*_*ij*_ and *α*_*ij*_, mutation changes the trait value according to a truncated normally distributed random variable (with variance *σ*), centred around the current trait value. Mutation occurs on these traits with probabilities *μ*_p_ and $\mu_{\alpha }$, respectively. We assume a higher mutation rate for *p*_*ij*_ than for the other traits. Asocials carry the *p*_*ij*_ and *α*_*ij*_ traits, but do not express them.

### (c) Individual fitness

Following [20], the expected number *w*_*τj*_(*t*_g_) of offspring that survive to adulthood (fitness) produced by an individual of type *τ* ∈ {a, c, d} (asocial, cooperator and defector, respectively) in patch *j* at time *t*_g_ (where the subscript g denotes generational time) is assumed to follow a discrete time Beverton–Holt model (e.g. [41]) with two niches, social and asocial. The degree of competition between these niches is set by two parameters, *η*_as_ and *η*_sa_, which give the *per capita* effect of socials on asocials’ fitness, and asocials on socials’ fitness, respectively. According to these assumptions, we write the fitnesses of the three types on patch *j* at time *t*_g_ as

2.1 $$\begin{array}{rl} & w_{aj}(t_{g})=\frac{r_{a}}{1+n_{aj}(t_{g})/K_{a}+\eta_{\mathrm{as}}[n_{cj}(t_{g})+n_{dj}(t_{g})]},\\ & w_{cj}(t_{g})=\frac{r_{cj}(t_{g})}{1+[n_{cj}(t_{g})+n_{dj}(t_{g})]/K_{sj}(t_{g})+\eta_{\mathrm{sa}}n_{aj}(t_{g})}\\ \mathrm{and}\; & w_{dj}(t_{g})=\frac{r_{dj}(t_{g})}{1+[n_{cj}(t_{g})+n_{dj}(t_{g})]/K_{sj}(t_{g})+\eta_{\mathrm{sa}}n_{aj}(t_{g})}, \end{array}\}$$

where *n*_*τj*_(*t*_g_) is the number of individuals of type *τ* on patch *j* at time *t*_g_. The numerator in each expression can be thought of as the maximal growth rate of an individual of the corresponding type, while the denominator as the intensity of density-dependent competition faced by that individual. This Beverton–Holt fitness model is demographically explicit, and so allows us to capture the feedback between group size and cooperation, institutional rules and hierarchy.

We now detail the parameters in the above fitness functions.

### (d) The economic game: cooperation and institutionally arranged punishment

First, *r*_a_ (≥0) is the maximal growth rate of an asocial type. The maximal growth rate of a cooperator is assumed to be given by

2.2 $$r_{cj}(t_{g})=r_{a}-\iota_{j}(t_{g})-C,$$

where $\iota_{j}(t_{g})$ (≥0) is the cost of participating in deciding institutional rules via consensus formation (defined below), while *C* (≥0) is the individual cost of producing an amount *B* of public good. This entails that an amount *n*_c*j*_(*t*_g_)*B* of public good is created on patch *j* by cooperators, which can be devoted to resource enhancement via production of technology, or to monitoring and punishment of defectors. Social defectors participate in institution formation but do not contribute to the public good, and can be punished for this. We assume that the maximal growth rate of a defector is

2.3 $$r_{dj}(t_{g})=r_{a}-\iota_{j}(t_{g})-\frac{\;p_{j}(t_{g})n_{cj}(t_{g})B}{n_{dj}(t_{g})},$$

where *p*_*j*_(*t*_g_) is the proportion of the public good produced on patch *j* devoted to punishment.

The remainder of the public good not used for punishment, (1 − *p*_*j*_(*t*_g_))*n*_c*j*_(*t*_g_)*B*, is used to increase the carrying capacity of social individuals (both cooperators and defectors), *K*_s*j*_, above the baseline carrying capacity of asocials (a fixed parameter *K*_a_):

2.4 $$K_{sj}(t_{g})=K_{a}+\beta [1-\exp(-\gamma (1-p_{j}(t_{g}))n_{cj}(t_{g})B)].$$

This gives a maximal possible increase in carrying capacity of *β*. The parameter *γ* sets the gradient of the increase in carrying capacity with respect to investment of the public good in technology.

### (e) The political game: setting institutional rules via consensus formation

To complete the model specification, we need to specify how *p*_*j*_(*t*_g_) is formed from the preferences *p*_*ij*_(*t*_g_) of social individuals, i.e. the political game, and to specify the cost to social individuals $\iota_{j}(t_{g})$ arising from doing this. To do this, we use a standard consensus formation model [30,31,34].

Each social individual *i* has a current *opinion*, *x*_*ij*_(*t*_p*j*_), about what the value of *p*_*j*_(*t*_g_) should be on their patch, where *t*_p*j*_ represents a time step in the political game on patch *j* (i.e. within a generation *t*_g_). At the start of the political game *x*_*ij*_(0) = *p*_*ij*_(*t*_g_), i.e. each individual starts out with their own intrinsic preference that they inherited from their parent. Through discussing with other social individuals on their patch, they may then modify their current opinion *x*_*ij*_(*t*_p*j*_) away from their inherited preference, *p*_*ij*_(*t*_g_), until a consensus for the institutional rules *p*_*j*_(*t*_g_) is reached.

At each time step *t*_p*j*_ of the political game, one social individual is chosen to be a *speaker*, who talks to multiple other social individuals on the patch known as *listeners*. The probability *ρ*_*ij*_(*t*_p*j*_) of individual *i* on patch *j* to be chosen as a speaker is an increasing function of its *α* value as follows:

2.5 $$\rho_{ij}(t_{pj})=\frac{s_{ij}(t_{g})\alpha_{ij}{\left(t_{g}\right)}^{\lambda }}{\sum_{i=1}^{n_{j}(t_{g})}{\left(s_{ij}(t_{g})\alpha_{ij}(t_{g})\right)}^{\lambda }},$$

where *s*_*ij*_(*t*_g_) = 1 if individual *i* on patch *j* is social, and 0 otherwise, and *n*_*j*_(*t*_g_) = *n*_c*j*_(*t*_g_) + *n*_d*j*_(*t*_g_) + *n*_a*j*_(*t*_g_) is the total number of individuals on patch *j*. Social individuals with high *α* values compared to the mean are thus *leaders*—they are more likely to be selected to be a speaker, i.e. they are more talkative. Social individuals with lower *α* values are then *followers*. The degree of *hierarchy* is measured by positive skew of the distribution of *α* among social individuals on the patch. The skewness is measured by Pearson’s moment coefficient of skewness.

The exponent *λ* defines how much the difference in influence is translated into a difference in the probability to speak. Following [34], throughout this paper we set *λ* = 4 so that in a group of 1000 individuals with the most extreme hierarchy (one individual with very high *α* and the rest with very low *α*), the probability that a leader is chosen as a speaker is very high (close to 90%).

The speaker talks with *N*_l_ listeners at time *t*_p*j*_, sampled at random from among the other social individuals on the patch. During this discussion event, each listener v updates its opinion to a value following the equation below, where v represents the listener and u the speaker:

2.6 $$x_{v}(t_{pj}+1)=x_{v}(t_{pj})+(\alpha_{u}-\alpha_{v})(x_{u}(t_{pj})-x_{v}(t_{pj})).$$

How much the listener is influenced by the speaker depends on the difference in their *α* values. We also assume that the position of speaker gives a slight influential advantage over the listeners. Therefore, the minimum difference of influence *α*_u_ − *α*_v_ is set to a positive low value (0.01). At the next time step, *t*_p*j*_ + 1, an individual is again chosen to be the speaker according to equation (2.5) (which may be the same individual chosen in the previous time step, e.g. if there is a high positive skew of *α* on the patch). *N*_*l*_ social individuals are then chosen to be listeners, and update their opinions according to equation (2.6).

At the end of each time step, the following condition is evaluated, and the political game ends if it is true:

2.7 $$\sigma_{x}(t_{pj})<x_{\mathrm{thr}}.$$

This condition says that consensus is reached when the standard deviation of the opinions of social individuals *σ*_*x*_(*t*_p*j*_) is less than a threshold *x*_thr_. The number of discussion events that occurred to reach consensus is called the consensus time, $t_{pj}^{*}$. The value of *p*_*j*_(*t*_g_) agreed by the individuals is then the mean of the current *opinions* at consensus of the social individuals on the patch:

2.8 $$p_{j}(t_{g})=\frac{1}{n_{cj}(t_{g})+n_{dj}(t_{g})}\sum_{i=1}^{n_{\;j}(t_{g})}s_{ij}(t_{g})x_{ij}(t_{pj}^{*}).$$

The cost of playing the political game, $\iota_{j}(t_{g})$, then depends upon the number of time-steps taken to reach consensus, $t_{pj}^{*}$, as follows:

2.9 $$\iota_{j}(t_{g})=It_{pj}^{*},$$

where *I* is a parameter that scales the consensus time into a growth rate cost in equations (2.2) and (2.3).

Finally, we note that although individuals modify their expressed opinion *x*_*ij*_ during the political game to try to reach consensus, they still pass on their original preference *p*_*ij*_ to their offspring. In other words, although individuals may compromise in the political game, they still have their own preferences for what they would have liked the outcome to be, and these evolve on a slower time scale (over generations in our model). This assumption is supported by work in political science which (i) recognizes that choices that are made in collective decision-making circumstances can be different from individual preferences that might be made in isolation [42], and (ii) provides evidence that political attitudes are shaped via both genetic and environmental processes of inheritance from parents-to-offspring [43].

## 3. Results

Owing to the strong nonlinearity of our model, we analyse the stochastic process by means of individual-based simulations. The baseline parameters used for the simulations, unless otherwise specified, are given in table 1. In all simulations, we start the population with 100% asocial individuals, with their carrying capacity of *K*_a_ = 20. These initial individuals have random generated values for their *p*_*ij*_ and *α*_*ij*_ traits, so that the population starts with no particular preference for the institutional rules, and no hierarchy. We then determine conditions under which social individuals invade and establish institutional rules that select for cooperation and increase in group size (*K*_s*j*_), and examine the range of conditions where this leads to the evolution of hierarchy.

**Table 1.** Baseline parameter settings, chosen to match the original model [20] and previous work on consensus formation [34].

parameter	value
cost of cooperating, *C*	0.1
base growth rate, *r*_a_	2
base carrying capacity, *K*_a_	20
threshold variance in opinions to reach consensus, *x*_thr_	0.03
number of listeners during a discussion event, *N*_l_	30
difference in influence exponent, *λ*	4
consensus time cost scalar, *I*	0.01
*per capita* effect of asocial individuals upon socials, *η*_sa_	0.05
*per capita* effect of social individual upon asocials, *η*_as_	0.05
maximum increase in carrying capacity owing to cooperation, *β*	300
mutation rate on *τ*, $\mu_{\tau }$	0.01
mutation rate on *p*, *μ*_p_	0.02
mutation rate on *α*, $\mu_{\alpha }$	0.01
variance of normal distribution used for mutations on *p* and *α*, *σ*	0.1
number of patches, *N*_p_	50
benefit of cooperation, *B*	0.9
gradient of increase in carrying capacity, *γ*	0.0075
migration rate, *m*	0.1

**Figure 1.**
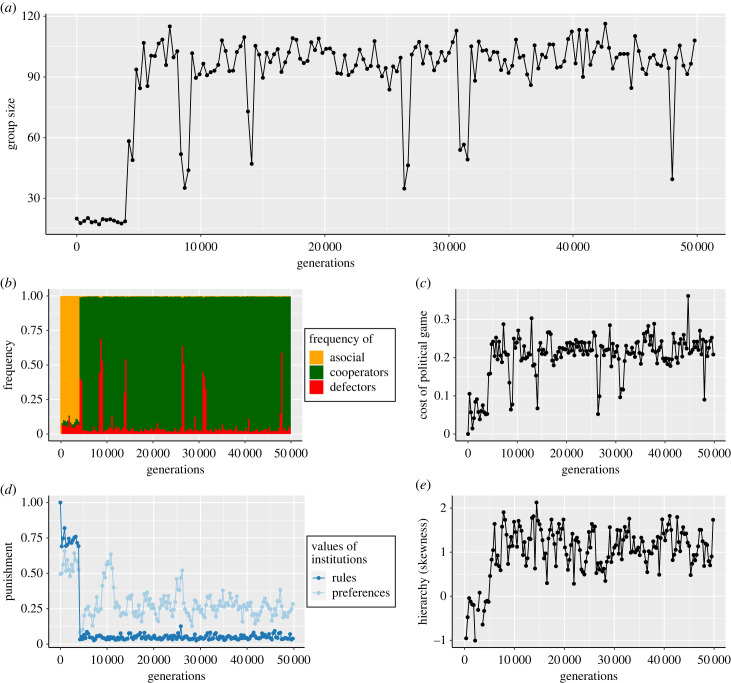
Illustration of the evolution of (*a*) group size, (*b*) frequency of each strategy, (*c*) cost of political game, (*d*) institutional rules and preferences, and (*e*) hierarchy measured by skewness, for the baseline model parameters in table 1. The results presented are means across either patches (*a*, *c*, *d* ‘rules’, and *e*), or all individuals in the population (*b* and *d* ‘preferences’), for a single run.

Figure 1 illustrates the dynamics of the model on a single run with the baseline parameters in table 1. The results show that socials invade a population of asocials (figure 1*b*). Moreover, we see both the evolution of cooperation (figure 1*b*), increasing group size (mean *K*_s*j*_ across patches, figure 1*a*), and the evolution of hierarchy (increased skew in *α*_*ij*_ on patches, figure 1*e*). This happens because if social individuals cooperate, they will outcompete asocial individuals on their patch. This is because they will produce a public good that is used to increase their carrying capacity (equation (2.4)) above that of asocials (*K*_a_), which then leads to suppressed reproduction and ultimately extinction of asocials on their patch (equation (2.1)).

However, why is cooperation then favoured rather than defection among socials? Cooperation will be favoured when *r*_c*j*_(*t*_g_) > *r*_d*j*_(*t*_g_). From equations (2.2) to (2.3), we can see that this is when the cost of cooperating is less than the cost of being punished for defecting [44], i.e. when *C* < *p*_*j*_(*t*_g_)*n*_c*j*_(*t*_g_)*B*/*n*_d*j*_(*t*_g_). This crucially depends on the institutional rules, i.e. the value of *p*_*j*_(*t*_g_). Figure 1*d* shows that groups evolve, via the preferences of social individuals *p*_*ij*_, to invest some of their public good into punishment, allowing the condition to hold true. However, successful groups do not invest too much into punishment, as this is inefficient. The most successful groups invest just enough into punishment to incentivise cooperation in their group, and use the remainder to increase their carrying capacity and hence the number of offspring their members have (equation (2.4)). These groups grow to a larger size, and so export a larger number of migrants to other groups. The migrants carry their preferences for their successful institutional rules, *p*_*ij*_ with them to their new groups, and so bias other groups towards having the same rules.

The evolution of hierarchy occurs because the cost of playing the political game (figure 1*c*), $\iota_{j}(t_{g})$, depends on the time taken for the group to reach consensus on the institutional rules, $t_{pj}^{*}$ (equation (2.9)). The time taken to reach consensus in this standard consensus formation model increases with group size [34]. Moreover, [34] demonstrates that in this model the rate of increase in consensus time with group size depends upon the skew of *α*_*ij*_ values in the group. Groups with one or a small number of leaders—individuals with *α* values much higher than the rest of their group—increase their consensus time at a slower rate as their group size increases, compared to egalitarian groups which do not have this skew in the *α* traits of their members. As groups evolve cooperation-promoting institutions and grow in size from the benefits of cooperation, this creates selection pressure for hierarchy in order to reduce $\iota_{j}(t_{g})$ (the mean value of this across groups is shown in figure 1*c*). Figure 1*e* shows that hierarchy evolves as groups start to create institutions (figure 1*d*) and increase in size (figure 1*a*). As groups increase in size, social individuals in groups with hierarchy will have more offspring than social individuals in groups without hierarchy (equations (2.2) and (2.3)). This causes a spread of a positively skewed distribution of *α* to different patches, with a minority of individuals evolving to act as leaders, and the rest as followers.

As a control, we ran a version of the model where it was not possible for hierarchy to evolve. To do this, we initialized the population so that every individual had *α* = 0.5, and then did not allow mutations on this trait. In this case, social individuals were unable to form cooperation-promoting institutions as their group size increased. Specifically, in the control the mean frequency of cooperation over 50 000 generations and 10 replicates was 0.14, compared to 0.91 in the base model. Likewise, the average group size (*K*_s_) was 28 in the control, compared to 147 in the base model. This demonstrates that in the model hierarchy is necessary to allow groups to maintain cooperation-promoting institutions as they grow in size, in order to reduce the cost of creating institutional rules via the political game of consensus formation.

An important effect of hierarchy is that the institutional rules reached by consensus, *p*_*j*_(*t*_g_), are biased in favour of the preferences of leaders. This follows from equation (2.6), where how much a listener changes their opinion depends on the difference in the *α* values of the speaker and listener. Moreover, leaders with high *α* values are more likely to be chosen as speakers in the first place (equation (2.5)). The ‘punishment’ graph in figure 1*d* shows the average (mean across patches) difference between the mean value of the preference *p*_*ij*_ of individuals on the patch, and the actual value of the institutional rules, *p*_*j*_(*t*_g_), reached at consensus. This difference occurs because leaders’ *p* preferences have the biggest effect on the consensus, and yet there are only a few such individuals on each patch. For the majority of individuals, who are followers, their *p*_*ij*_ traits have much less effect on the consensus reached, and so they are under much less selection pressure. This allows them to drift to a certain degree. The electronic supplementary material, figure S1 shows the correlation between the *α*_*ij*_ and *p*_*ij*_ traits. The extent to which the consensus rules are dominated by the preferences of leaders is controlled by the parameter *x*_thr_, which we discuss in the next section.

Finally, figure 1*b* shows that there are occasional spikes of defection. These occur when groups start to invest too little into punishment. Specifically, if a group has only cooperators then it can temporarily do very well by investing nothing into punishment and everything into increasing carrying capacity. This causes the group to grow to a larger size, and send out more migrants to other patches. However, once defectors arise either by mutation or migration they can quickly spread in such groups, causing the group to in turn lose the benefits of cooperation and hence reduce in size. At this point, groups evolve to invest more in punishment again, as migrants from more successful groups arrive with these preferences. Usually this process occurs rapidly, and so the global decline in cooperation is small and temporary. Occasionally, however, several groups can evolve to invest too little into punishment at the same time. In this case, it takes longer for punishment to be restored by migration from other groups, causing a temporary global decline in punishment and cooperation.

### (a) The effect of the threshold necessary to reach consensus, *x*_thr_

The parameter *x*_thr_ specifies how close the opinions of social individuals have to be before consensus is reached, the political game ends, and *p*_*j*_(*t*_g_) is set for their group (equation (2.7)). It thus affects the time to reach consensus, and hence the cost of playing the political game. However, it also affects the extent to which the consensus differs from the mean preference of the individuals. If *x*_thr_ = 1 then consensus is reached immediately, and so *p*_*j*_(*t*_g_) is then given by the mean *p*_*ij*_ of all social individuals on the patch. Conversely, the lower the value of *x*_thr_ then the greater the number of discussion events that need to take place before *σ*_*x*_(*t*_p*j*_) < *x*_thr_ and consensus is reached. At each discussion event, leaders are more likely to be chosen to speak than followers, and so the opinions of individuals are more likely to shift towards that of a leader than that of a follower. The more discussion events there are, the more times this shift in the opinion of followers is likely to occur, and so the agreed investment into punishment *p*_*j*_(*t*_g_) at consensus will be closer to the preferences of leaders rather than followers.

Empirically, we can think of *x*_thr_ as representing how difficult the political game is, i.e. how close the opinions of individuals have to be before they will accept the consensus rules. A lower value of *x*_thr_ represents a more difficult political game. This would correspond to the political game producing rules for an economic game that is very important for the material pay-off of group members, and so each group member has a high stake. A larger value of *x*_thr_ represents an easier political game, i.e. rules that have relatively little effect on the material pay-off of group members.

**Figure 2.**
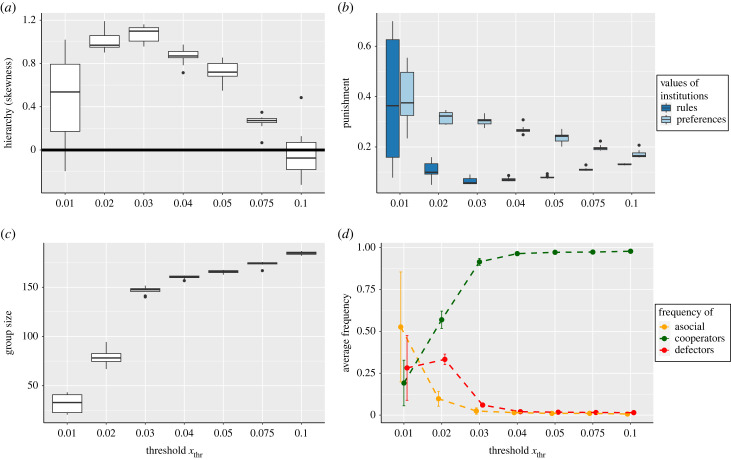
The effect of varying how difficult the political game is, as determined by the threshold variance in individual opinions necessary to reach consensus, *x*_thr_ on (*a*) hierarchy, (*b*) institutional rules and individual preferences, (*c*) group size and (*d*) frequency of each strategy in the population. Lower *x*_thr_ represents a more difficult political game, in which individuals have a higher stake and so find it more difficult to reach consensus. The boxplots represent measures across 50 000 generations and 10 replicates, and are over patches. Error bars in plot (*d*) represent the standard deviation.

Figure 2 shows the effect of varying *x*_thr_ on the evolution of institutional rules (*b*), cooperation (*d*), group size (*c*) and hierarchy (*a*). For intermediate values of *x*_thr_, we find the same results as in figure 1—social individuals invade a population of asocials (*d*) and establishing cooperation-promoting institutions (*b*), which lead to increasing group size (*c*) and hierarchy (*a*). For high values of *x*_thr_, cooperation-promoting institutions evolve and drive an increase in group size, but we no longer see the evolution of hierarchy. In these cases, the time to reach consensus is fast even without hierarchy, leading to a low cost of playing the political game (equation (2.9)) even in large egalitarian groups. There is thus not enough benefit to having hierarchy on the political game to lead to the evolution of a skewed distribution of *α*_*ij*_. On the other hand, for very low values of *x*_thr_ groups are unable to evolve cooperation-promoting institutions.

**Figure 3.**
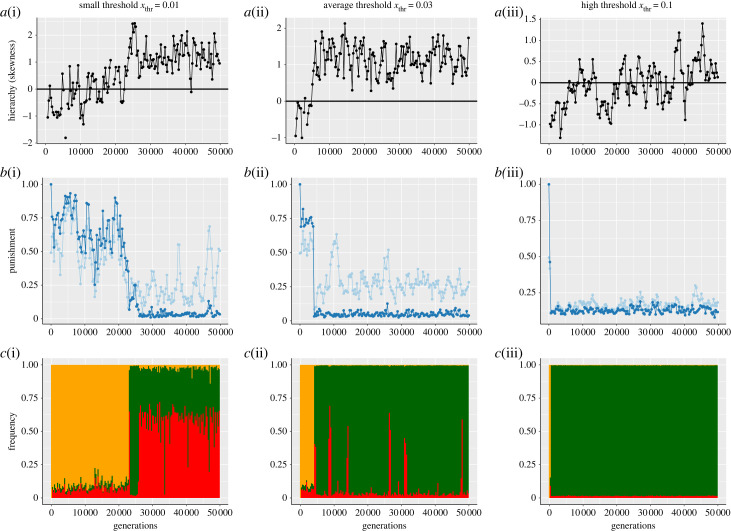
Illustration of the evolution on a single run of (*a*) hierarchy (mean across patches), (*b*) institutional rules (dark blue, mean across patches) and individual preferences (light blue, mean across all individuals in the population), and (*c*) frequency of each strategy in the population, for (i) hard political games, (ii) medium political games and (iii) easy political games (low, average and high *x*_thr_, respectively).

Figure 3 illustrates the dynamics of evolution during a run, and shows why this is the case. First, with a very low value of *x*_thr_, the time to reach consensus is high even in small groups. This makes it more difficult for social individuals to invade a population of asocials (figure 3*c*(i)). More formally, the expected waiting time for socials to invade asocials in the stochastic simulations increases as *x*_thr_ becomes smaller. This effect is illustrated in the ‘frequency’ graphs (figure 3*c*, row). Second, once social individuals have invaded then for smaller values of *x*_thr_ they need more discussion events to reach consensus. As discussed above, the more discussion events there are, the more the rules reached at consensus are biased towards the preferences of a small number of leaders. This means that the preferences of followers matter less and so are subject to drift, as illustrated in the ‘punishment’ graph (figure 3*b*(i)). However, this drift in the preferences of the majority of group members then weakens competition between groups, which occurs via differential migration. In the baseline model, [20], individuals in groups that invest just enough into punishment to incentivise cooperation produce more offspring, and then export their preferences for these ‘optimal’ institutional rules to other groups. This mechanism functions effectively when migrants have an effect on the institutional rules in their new groups. This was the case in [20], where the institutional rules were formed by taking the mean preference of the group members. This is also the case for medium and high *x*_thr_ in our model. However, for low *x*_thr_, followers—who make up the majority of migrants—have little effect on the consensus, and their preferences drift. This dilutes competition between the institutions of different groups, and leads to groups choosing rules that invest too little into punishment to incentivise cooperation. When the majority of individuals have little to no say in the institutional rules then the group loses the benefits of the ‘wisdom of the crowd’ [25], and so can fail to move to more optimal rules that would maintain higher levels of cooperation.

### (b) Sensitivity to parameters

The electronic supplementary material, figure S2 shows the effect of varying the benefit of cooperation, *B*. For low *B*, institutional rules are selected that invest a greater proportion of the public good into punishment. However, this greater investment leads to a smaller increase in *K*_s_ and hence a smaller group size. On the other hand, for a larger *B* a smaller proportion of the public good is invested into punishment—but this smaller proportion is still larger in absolute terms and so can maintain cooperation while allowing groups to grow even larger. The institutional rules thus evolve to match the efficiency of cooperation.

The effects of varying the initial group size, *K*_a_, were studied in [20]. This must be small to allow social individuals to invade a population of asocials. However, once socials have invaded, their institutionally supported punishment can maintain cooperation as groups become much larger. The consensus time cost scalar, *I*, is multiplied by the consensus time to translate the time into a fitness cost. If this is 0 then cooperation-promoting institutions evolve without hierarchy. Conversely, if it is very large then cooperation-promoting institutions cannot evolve, because the fitness cost of having an institution is too large. The number of listeners during a discussion event, *N*_l_, affects how quickly a group reaches consensus. For larger *N*_l_, consensus is reached quicker [31]. Empirically, we would expect *N*_l_ to be larger if groups have more effective communication technology (e.g. writing), or if the political game is itself structured to facilitate opinion formation, e.g. through individuals meeting in organized assemblies to exchange views.

## 4. Discussion

Our model demonstrates that, under a wide range of conditions, the evolution of hierarchy can result from the need to facilitate the implementation of cooperation-promoting institutions. As groups grow in size, owing to the benefits of their cooperative activities, then the process of negotiating and forming their institutional rules—their political game—becomes more costly. This scalar stress [16] favours the evolution of hierarchy to reduce the cost of rule formation.

Our model provides a plausible mechanism to explain the broad-scale trend of human social evolution, which has seen human societies get larger and more hierarchically structured [9,13–15], particularly since the development of agriculture [12,45,46]. More fine-scaled observations also support the predictions of our model. For example, archaeological information from Mesa Verde in the southwest United States documents an association between increasing settlement populations and increasing evidence of hierarchy [47]. Agriculture in the form of crop production is thought to be a particularly important driver of this trend owing to the fact it created a more sedentary lifestyle and increased population densities, thus increasing scalar stress and presenting new challenges to coordination and collective action that would require new institutional arrangements [48–51]. However, evidence from non-agrarian contexts also supports our model. For example, comparative evidence from nomadic pastoralists shows that societies with larger camp sizes are associated with greater degrees of social hierarchy [16]. Furthermore, von Rueden & van Vugt [52] present ethnographic examples from hunter–gatherer societies, which indicate that leadership roles became more prominent during periodic increases in population density. In the Yahgan of Tierra del Fuego, leaders emerged when people congregated to feed on whales, and helped coordinate activities and enforced order, while in Plains Indian bands summer aggregations for buffalo hunting saw the temporary election of a chief to oversee proceedings and police violations of rules.

Numerous hypotheses have been proposed to explain the evolution of hierarchy in human societies. It is beyond the scope of this paper to cover all these different ideas, or to assess whether our model is better supported empirically than others (see Currie & Perret [53] and Smith *et al.* [54], this volume for recent overviews). However, an important contrast is often made between those theories that focus on the benefit of hierarchy to the group as a whole (‘functional’, ‘managerial’ or ‘integrative’ theories, of which our model is an example), and those that focus on the benefits to those at the top of the hierarchy (‘extractive’, ‘despotic’, ‘conflict’ theories, e.g. Perret & Currie [55]). Our approach explicitly invokes the need to establish and modify rules as a key organizational problem that leads to hierarchy. There are many aspects to the process of rule formation, and in modern countries we are used to the idea that there are distinct executive, judicial and legislative aspects of governance. In smaller-scale societies, these distinctions may not be so formalized or separated, and indeed the political game in our model is abstract enough to cover the processes of consensus that occur across these domains. As such our approach potentially provides a more general framework that can unite other ‘functional’ theories for the evolution of leadership and hierarchy [56–60], which can be thought of as examples of playing a political game to establish effective rules (e.g. deciding rules over construction of irrigation systems [51,61–63], harvesting of marine resources [26,64,65], or defensive warfare [66,67]).

Our model focuses on the number of people involved in the political game, and only considers one rule on which consensus formation occurs. However, it is also important to note that as the size of a group increases, the number of rules proliferates. For example, the small-scale Kapauku Papuan society has around 120 rules regulating areas from property rights through to punishment for murder. Although this number could be pushed up if several other informal norms that guide behaviour in different contexts are included, it is still orders of magnitude smaller than that seen in modernized countries, e.g. 40 000 new laws took effect in the USA in 2014 alone [4]. Although we do not explicitly model different rules, we can speculate that having multiple rules on which consensus needs to be reached has a similar effect to having more difficult political games (i.e. *x*_thr_ is smaller). This potentially has implications for the nature of hierarchy as it is captured in our model, and as it plays out in the real world. In our model, individuals evolve to become more like leaders or more like followers based on their individual traits. This type of ‘achieved’ leadership is well-documented ethnographically in small-scale societies [52]. However, as our results show, this informal leadership has limitations as the political game becomes more difficult (i.e. when *x*_thr_ becomes smaller). Formal leadership, where there is a specific designated leader (or leaders) in a group, may overcome this. For example, a designated leader may have more time to devote to spend on thinking about what effective rules can be, or assessing how well existing rules are playing out or being observed. Also, a designated leader may learn rules from the leaders of other successful groups, avoiding the need for successful rules to spread via differential migration, with the problems that entail for difficult political games. The flip slide, though, is that more formal leadership may lead to economic inequality within groups, with leaders monopolizing the group’s resources for themselves and their kin. We have not explicitly considered economic inequality in our model. Other models have studied this in the context of leaders helping to solve coordination or collective action *economic* games, and then taking a (possibly disproportionate) share of the benefits [59,68,69]. These existing models have not, though, studied the role of leaders in reducing the cost of playing *political* games. As societies grew larger they did indeed formalize leadership into a hereditary position (chiefdoms), and ultimately multiple layers of hierarchy and more specialized bureaucratic offices (states) [70]. In future, these kinds of changes in the nature of hierarchical relationships can be explored by extending the meta-model approach used here further to include a ‘constitutional’ game, which establishes the conditions under which the political game takes place, including who gets to take part [10,23,71].

## Data Availability

The code is available from the Github repository: https://github.com/simon-powers/Co-evolution-of-institutions-and-hierarchy. Information is also provided in the electronic supplementary material [72].
